# The Influence of Various Modifications of Hazelnut Shell Flour as Potential Filler in Plywood Technology

**DOI:** 10.3390/ma17164128

**Published:** 2024-08-20

**Authors:** Marta Wronka, Damian Wojnicz, Grzegorz Kowaluk

**Affiliations:** 1 Faculty of Human Nutrition, Warsaw University of Life Sciences—SGGW, Nowoursynowska St. 159, 02-776 Warsaw, Poland; s223012@sggw.edu.pl; 2 Institute of Wood Science and Furniture, Warsaw University of Life Sciences—SGGW, Nowoursynowska St. 159, 02-776 Warsaw, Poland

**Keywords:** plywood, binder, filler, hazelnut shell, residue, modification, upcycling

## Abstract

This study investigates the potential of utilizing hazelnut shells (HS) as an innovative filler in three-layer plywood technology, addressing the growing need for sustainable, high-performance materials. Traditional plywood production relies on adhesives enhanced with various fillers to improve physical, mechanical, and operational characteristics. This research explores using native, chemically modified, and activated carbon derived from hazelnut shells as fillers in urea–formaldehyde (UF) resin. The produced plywood’s mechanical properties, water absorption, and formaldehyde emissions were thoroughly analyzed. Key findings demonstrate that incorporating 10 part by weight (pbw) native hazelnut shell flour significantly enhances the modulus of rupture (MOR) to 138.6 N mm^−2^ and modulus of elasticity (MOE) to 13,311 N mm^−2^. Chemically modified hazelnut shell flour achieves optimal results at 5 pbw, while activated carbon from hazelnut shells, even at 1 pbw, markedly improves bonding strength (2.79 N mm^−2^ referred to 0.81 N mm^−2^ for reference sample without filler added). Notably, activated carbon effectively reduces formaldehyde emissions (2.72 mg 100 g^−1^ oven dry panel referred to 3.32 mg 100 g^−1^ oven dry panel for reference samples with 10 pbw filler) and improves water resistance, indicating better further dimensional stability and lower environmental impact. The study also shows that excessive filler content negatively affects strength parameters, confirming the importance of optimizing filler concentration. These results highlight the potential of hazelnut shells as an eco-friendly alternative filler in plywood production, contributing to waste valorization and environmental sustainability. This study supports the practical application of hazelnut shell fillers, promoting a circular economy and reducing reliance on traditional, less sustainable materials, thus providing a valuable solution for the wood composite industry.

## 1. Introduction

The plywood production technology has remained unchanged for over a century, but ongoing research aims to enhance its quality. The primary advancements in improving plywood involve adopting modern equipment and novel adhesives. On a daily basis, the furniture business utilizes adhesives with various fillers to produce plywood, which improves its physical–mechanical, technological, and operational features. Research is focused on generating adhesives with fewer formaldehyde emissions and using renewable resources, signaling a move toward non-formaldehyde or low-emission formaldehyde-based resin for sustainable and equivalent adhesive solutions [1,2]. Fillers are characterized according to numerous parameters such as size, role, dimensions, source, and morphology [3]. Fillers are divided into organic and inorganic [4]. The first category includes components including wood flour, starch, wheat flour, soya flour, and lignin. Inorganic fillers include nanoparticles of various chemicals such as titanium dioxide and zinc [4] or kaolin [5]. Inorganic fillers, such as precipitated calcium carbonate (PCC), provide improved mechanical characteristics and greater replacement ratios, contributing to the increased mechanical strength in the polymer composites [6]. Organic fillers swell in water, actively absorb moisture, enhance adhesive viscosity, and minimize veneer glue dripping [7]. Biomaterials using natural fillers, such as bagasse, eggshell, and lemon leaves, have demonstrated superior mechanical properties, including strength under tension, resistance to indentation, and mass per unit volume, making them appropriate for various uses [8,9]. Inorganic–organic hybrids are frequently employed as fillers for polymer composites, having better physicochemical, thermal, or mechanical qualities [10]. Beech bark and corn cob powder were investigated as eco-friendly fillers for UF adhesives in plywood manufacture, demonstrating good benefits on mechanical characteristics and formaldehyde emissions [11,12]. Also, walnut shells can be mentioned as a widely available and low ash raw material that can be successfully applied in wood bonding by conversion into lignin–formaldehyde resin [13]. The application of modified corn cob powder as a filler for UF resin in plywood manufacturing successfully stopped the UF adhesive from curing prematurely and greatly lowered its viscosity, resulting in increased bonding strength [14]. Oak and birch bark were also investigated as suitable fillers, demonstrating lower formaldehyde emissions and enhanced bonding quality in plywood manufacture [15]. Wood flour as a filler in plywood composites showed a drop in tensile strength and impact strength with an increase in filler concentration but a beneficial effect on the modulus of elasticity at lower binder content [16]. The investigation of using various bark species as fillers for UF resin in three-layer plywood manufacturing revealed decreased tensile strength and varied formaldehyde emissions. The results showed that the type of bark filler employed had an influence on the strength metrics and formaldehyde emissions of the resulting plywood [17,18], supporting the practical growth of a circular economy and assisting in environmental pollution control [19]. Life cycle assessment (LCA) studies have demonstrated that the use of organic fillers in the plastics industry can assist in minimizing emissions to the environment, highlighting the environmental benefits of employing bio-based fillers [20,21]. This research implies possible economic benefits in terms of increased adhesive qualities and lower emissions. On the other hand, inorganic fillers, specifically when used in large amounts, may have a greater environmental impact, especially when considering end-of-life treatment and recycling procedures [22].

The processing of nuts, which are widely available in stores, generates shells, which are typically discarded, but to avoid wasting the potential of such raw materials, for example, cashew nut shells (CNSs) are converted into solid briquettes [23]. Cashew nut shells can also be used to generate activated charcoal [24]. Nutshells such as pistachio shells [25], palm nut shells [26], and Brazil nut shells [27] were used to produce activated carbon. As evident, scientists typically utilize products that are readily available in their specific regions. Activated carbon, generated from diverse waste materials, provides environmental benefits such as reduced material waste and a greater exploitation of natural resources [28]. Activated carbon is a carbon-based material that has been processed to be highly porous, offering a large surface area and diverse chemical functionalities [29]. It is produced by subjecting waste biomass or fossil resources like coal to high temperature and chemical activation [30,31]. Activated carbon production involves several methods, including pyrolysis activation, physical activation, chemical activation, and steam pyrolysis [32]. Activated carbon features a rigid carbon matrix with a high surface area and diverse functional groups, enabling it to attract and bind various molecules in both gas-phase and liquid-phase applications [29,30]. It is employed in a wide range of applications, such as purifying drinking water, air, and gas and medical treatments for poisoning and overdoses. Additionally, it is used in industrial processes for the removal of contaminants, in air filters for environmental control, and in the food and beverage industry for decolorization, purification purposes [33], the treatment of wastewater and leachate [32], and soil detoxification from pesticide residue [34]. Materials made from silicone-modified activated carbon demonstrated resistance to combustion [35]. Activated carbon aerogels created from carboxymethyl cellulose can be effectively used in energy storage applications [36].

Activated carbon is also used in wood composite technology. It has been found that the addition of activated carbon to particleboards improves their mechanical properties, indicating better bonding and increased strength [37]. This study investigated the effect of incorporating different proportions of activated carbon (0%, 1.5%, 4.5%, 7.5%) on the properties of particleboard. The results demonstrate that the density increased with higher activated carbon content while the moisture content decreased, suggesting better dimensional stability and water resistance. Mechanical properties, such as the internal bond strength, modulus of rupture, and modulus of elasticity, significantly improved with the addition of activated carbon, indicating enhanced bonding and increased strength. Additionally, thermal conductivity decreased as the activated carbon content increased, leading to improved insulation performance. In summary, incorporating activated carbon at a ratio of 4.5% in particleboard significantly enhances its physical, mechanical, and thermal properties. Activated carbon was utilized as a filler in epoxy resin, resulting in smoother surfaces and enhanced characteristics [38]. The impact of activated charcoal on the curing kinetics and crosslink density of UF resin was studied using differential scanning calorimetry. The results revealed that activated charcoal accelerates the curing process of UF resin, increases the crosslink density, and reduces the activation energy. Notably, in medium-density fiberboard, the addition of activated charcoal enhanced the modulus of rupture and internal bond strength, indicating improved performance with even a small amount of activated charcoal. Additional benefits include that the activation energy of UF resin decreases with the increasing concentration of activated carbon, suggesting lower curing temperatures for the resin, and the crosslink density of UF resin improves significantly with activated carbon addition. Additionally, formaldehyde emissions significantly decreased by incorporating activated carbon [39,40].

Based on the literature review conducted, it can be stated that the conversion of hazelnut shells into activated carbon is not a widely adopted solution. However, experiments have used hazelnut shells and ground them into powder form in research on wood materials. Walnut shells and dregs were utilized as fillers in plywood with the dregs demonstrating better characteristics and fewer formaldehyde emissions than the shell filler [41]. Palm kernel meal and palm shells have been studied as fillers for wood adhesives. Their addition positively affected the shear strength in plywood [42]. Hemp flour and rye flour were both investigated as fillers for urea–formaldehyde resin. Hemp flour showed promise in reducing formaldehyde emissions without compromising the mechanical properties of plywood [43].

The global hazelnut yield was 1.0 tons per hectare in 2021, marking a 1.2% decline compared to the previous year [44]. Despite irregular yield fluctuations, the growth trend persisted during 2016–2021 with an average annual increase of 7.0%. The growth in the hazelnut industry is driven by increasing market demand. The hazelnut market is projected to grow from USD 474.21 million in 2023 to USD 700 million by 2028 with a compound annual growth rate (CAGR) of 8.1%. Demand for hazelnuts is rising due to consumer preferences for their beneficial health properties. The unused biomass after the shelling process, known as hazelnut shells (HSs), accounts for approximately 50–55% of the product weight in shells and is currently predominantly used as boiler fuel [45].

To summarize the literature review prepared, hazelnut cultivation worldwide is increasing, leading to a higher volume of waste in the form of shells. To prevent an increase in carbon dioxide emissions into the atmosphere through the incineration of this waste and simultaneously recognize the potential of activated carbon, the aim of this study was to apply the hazelnut shells in various stages (native, chemically treated/delignified, activated carbon from hazelnut shells) as a filler in three-layer plywood technology.

## 2. Materials and Methods

### 2.1. Materials

The rotary cut birch (*Betula* L.) veneer of an average thickness of 1.5 mm, 5 ± 1% moisture content (MC), and dimensions of 360 mm × 360 mm were used to produce plywood. As a binder, an industrial UF resin S-120 (Silekol Sp. z o.o., Kędzierzyn-Koźle, Poland) of about 65% of dry content [46] was used with ammonium nitrate water solution (industrial hardener; 20 wt%) as a hardener, pH about 6.2 ± 0.1, to reach the curing time of REF0 (Table 1) bonding mass at 100 °C in about 88 s. The rye flour was used as a reference filler. The mentioned UF resin was also the base of the tested bonding mixtures of different hazelnut shell flour fillers.

The hazelnut (*Corylus avellana* L.) shells were kiln-dried to the moisture content of about 3 ± 0.5%; then, they were ground in a Retsch SM 100 knife mill (Retsch GmbH, Haan, Germany) to a fraction below 0.7 mm. Such a fraction has been used or further processed as follows:

- Native: The powder has been sieved, and the fraction below 0.25 mm has been used in the research.
- Chemically modified: The hazelnut flour has been subjected to chemical components amount characterization according to the following methods: cellulose content—Seifert method, lignin content by TAPPI method, holocellulose content with sodium chlorite. The following results were achieved: 40.6% lignin, 28.8% cellulose, and 30.6% hemicellulose. The two-hour delignification process has been completed according to [47]. The final lignin content was 2.6%.
- Carbonized: The hazelnut shells flour has been subjected to heating in a laboratory prototype furnace under the following parameters: heating to 150 °C at a 10 °C ramp, heating to 300 °C at a 5 °C ramp, heating to 600 °C with a temp. ramp of 2 °C, storing at a constant temp. of 600 °C for 15 min and cooling down to room temperature. The fraction below 0.25 mm has been used in the research.

For the filler water absorption test, 3 filter paper containers were used per every tested filler. Approximately 1–2 g of tested filler was placed in each container. Three repetitions were performed for each variant. Each sample was soaked for 10 min in demineralized water at approximately 20 ± 1 °C, which was followed by 10 min of free draining. After this process, the weight of the samples was measured.

### 2.2. Preparation of Panels

A three-layer plywood was created using reference or alternative fillers. The glue mixture was manually and evenly applied to the veneers using a brush at a rate of 180 g m^−2^. Once the veneers were aligned correctly and joined, the samples were pressed using a high-temperature press (AKE, Mariannelund, Sweden) at 140 °C and a maximum unit pressure of 1.2 MPa for 7 min. The produced boards were conditioned before the tests at 20 °C and 65% ambient air humidity until a constant mass had been obtained.

### 2.3. Characterization of the Elaborated Panels

The test specimens were cut on a saw blade as required by European standards EN-326-2 [48] and EN-326-1 [49]. The modulus of rupture (MOR) and modulus of elasticity (MOE) were determined according to EN 310 [50], and the shear strength was measured according to [51]. All the mechanical properties were examined with a computer-controlled universal testing machine delivered by the Research and Development Centre for Wood-Based Panels Sp. z o. o. (Czarna Woda, Poland). The density profiles of the tested panels were measured on a GreCon DAX 5000 device (Fagus-GreCon Greten GmbH and Co. KG, Alfeld/Hannover, Germany). The formaldehyde (HCHO) emission has been determined according to [52] on two samples per tested panel, where one representative from every panel type has been selected to that test based on the highest mechanical properties (MOR) as well as an REF10 as reference panel. The number of samples of every tested panel was not less than 10 (3 for the density profile, 2 for HCHO emission) for every above-mentioned test. The density profile measurement results are the representative plots selected after analyses of 3 individual plots for every tested panel.

### 2.4. Statistical Analysis

Analysis of variance (ANOVA) and *t*-test calculations were used to test (α = 0.05) for significant differences between factors and levels, and where appropriate, using IBM SPSS statistic base (IBM, SPSS 20, Armonk, NY, USA). A comparison of the means was performed when the ANOVA indicated a significant difference by employing the Duncan test. The statistically significant differences in achieved results are given in Table 2 as homogenous (similar letter codes; no statistically significant differences) and non-homogenous (various letter codes; statistically significant differences occur) groups. The values on the plots, whenever possible, represent the mean values, and the error bars are the standard deviation values.

## 3. Results and Discussion

### 3.1. Modulus of Rupture and Modulus of Elasticity

**Figure 1:**
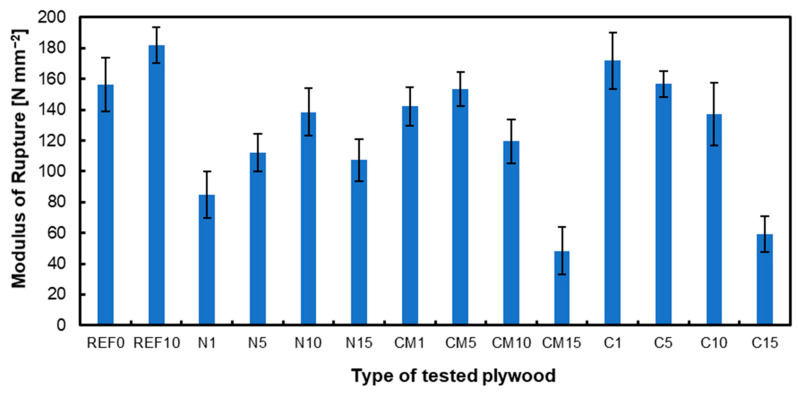
Modulus of rupture of tested panels.

**Figure 2:**
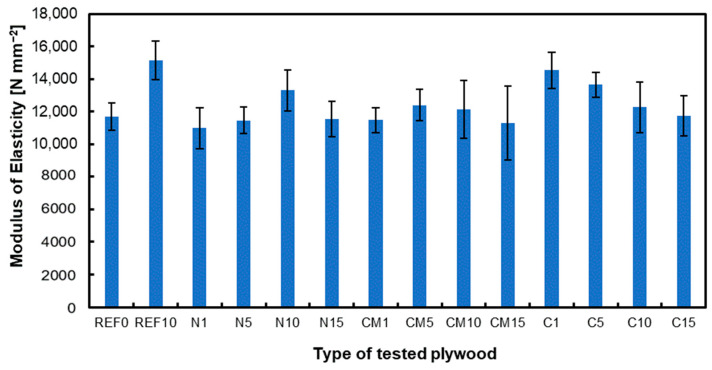
Modulus of elasticity of tested panels.

The MOR and MOE are presented in Figure 1 and Figure 2, respectively. The reference plywood containing 10% filler had the highest MOR values. When analyzing plywood with hazelnut shell milling dust, the optimum filler content was determined to be 10%, as higher filler content proved to be unfavorable. For plywood with chemically modified hazelnut shell dust, the optimum filler content was found to be 5% with higher proportions negatively affecting the MOR. The reason for improved parameters of the tested composite when applying chemically modified hazelnut powder can be the fact that without acetylation modification and additional NaClO processing, which is applied in most common delignification processes, the pores generated by removing more lignin could be more conveniently backfilled by the adhesive to eliminate the interface gap between the cell walls and the filled binder [47]. In the case of activated carbon from hazelnut shell dust, the best results were achieved with a filler content of 1%, which yielded the highest MOR values. Studies confirm that excessive filler content negatively impacts the strength parameters [17,53]. Including nanocellulose at specific concentrations improved bonding quality and plywood qualities, but higher amounts had a negative effect [54]. Activated carbon from coconut shells was incorporated into urea–formaldehyde glue for particleboard production. The glue was enhanced with 0.75% and 1% activated carbon. Based on the conducted tests, this additive positively influenced the mechanical properties of the panels [55]. The carbonization process enriches the carbon content and creates initial porosity, while the activation process enhances the pore structure, resulting in a large specific surface area. Therefore, even a small amount can significantly improve adhesive properties, such as viscosity [56]. Furthermore, it was found that using carbon nanotubes as a filler in electrically conductive adhesives substantially increases the viscosity of the adhesive, indicating the impact of carbon materials on the adhesive’s properties [57].

The MOE differences for the same samples show more minor variations between the variants because the wood mitigates the differences in joint stiffness with different types and amounts of filler. Studies confirm that several factors, such as the type of wood, adhesive, and joining method, can affect the MOE of the tested samples [58]. The wood species can also influence the MOE values [59].

### 3.2. Shear Strength

**Figure 3:**
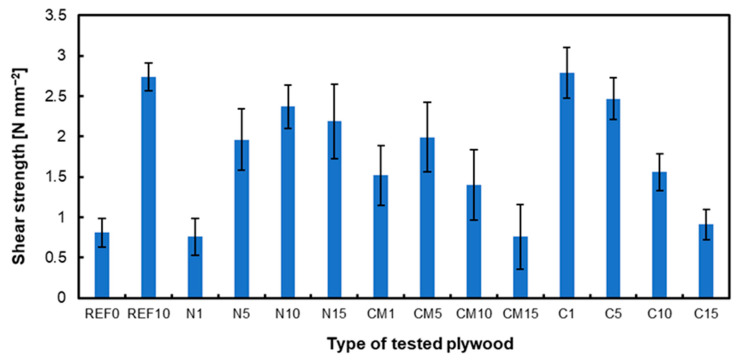
Shear strength of tested panels.

Figure 3 illustrates the shear strength of the composites studied. The results are consistent with those observed in MOR and MOE, highlighting more pronounced differences between the variants and the amounts of filler. Using alternative fillers in plywood technology, chestnut shell meal, walnut shells, and coffee waste were tested, each comprising 6% of the adhesive mass. Compared to the reference value, the shear strength improved significantly with walnut and chestnut shells [23]. However, with coffee waste, the shear strength decreased slightly. The results are consistent with those obtained using hazelnut shell dust. The selection of filler materials in plywood considerably impacts its shear strength. For example, using kenaf core powder and pine bark powder as adhesives in two-ply plywood has exhibited good bonding capacity, producing dry shear strengths of up to 1 MPa, exceeding the national standard for plywood [60]. The impact of activated carbon as a filler in wood composites, such as plywood, on mechanical properties has been studied. The addition of activated carbon filler affected the strength of the wood composites with medium-density fiberboard (MDF) samples exhibiting higher strength values than plywood samples due to the increased thickness of the activated carbon [61].

### 3.3. Water Absorption

**Figure 4:**
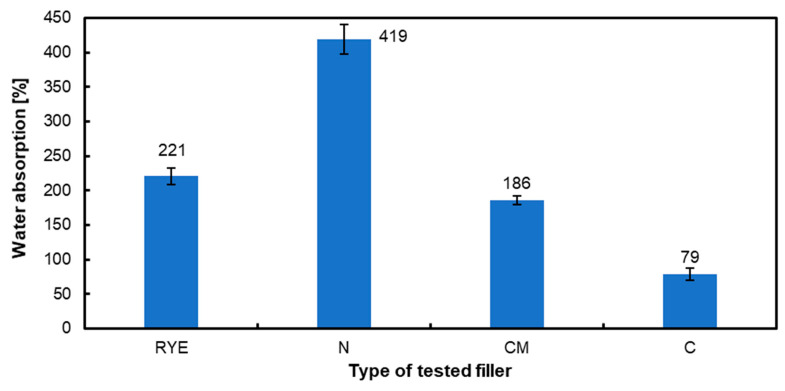
Water absorption of used fillers.

Figure 4 shows the adsorption capacity of the fillers used. The reference rye flour absorbed 221%, while the hazelnut shell flour absorbed 419%, which was the highest value obtained among the fillers used. For the chemically modified hazelnut shell flour, 186% absorption was obtained, and for the carbonized flour derived from hazelnut shell flour, the water absorption was the lowest at 79%. Such differences in water absorption can influence the mechanical properties of the tested composites, such as MOR and MOE. This is why the optimal, from the mechanical point of view (for example MOR), content of the filler was different for native (10 pbw), chemically modified (5 pbw) and carbonized (1 pbw). Water absorption depends on several factors, including the size of the particles or dust used, the composition of the material, and the immersion time [62,63,64]. Research reveals that activated carbon is an effective absorbent, but this is dependent on the raw material and activation process. The adsorption capacity of activated carbon is heavily influenced by the type of activation, whether physical or chemical [65]. The carbonization temperature, activation temperature, and water vapor flow all substantially influence activated carbon’s specific surface area and pore distribution [66]. The properties of activated carbon are strongly influenced by the raw material used to create it. For example, activated carbon produced from coconut shells and silica exhibited varying compositions and water content [67]. Activated carbon is often considered hydrophobic [68], which makes it hesitant to absorb water [65]. It is worth mentioning that just a small amount of activated carbon improved the mechanical qualities of the finished boards, whereas a larger amount reduced the strength parameters. Chemical modification does not always improve all examined properties, but it can exhibit other beneficial characteristics, such as lowering formaldehyde emissions. For instance, using modified fillers, such as corn cob powder and pine needle powder, decreased formaldehyde emissions, improving the environmental impact of plywood production [14,69].

### 3.4. Density Profiles

**Figure 5:**
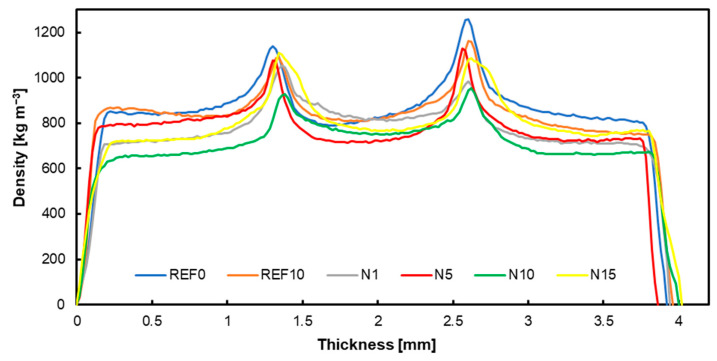
Density profile of tested panels with native hazelnut shell flour.

**Figure 6:**
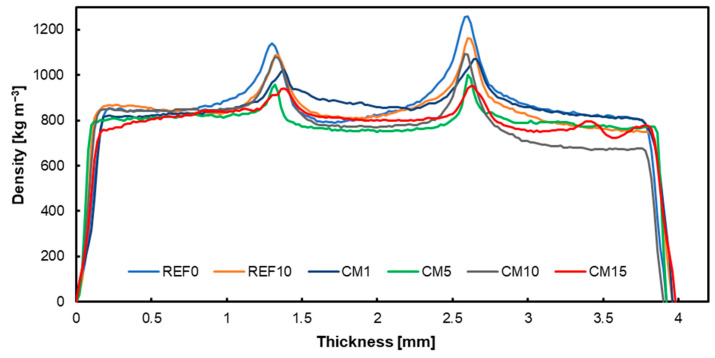
Density profile of tested panels with chemically modified hazelnut shell flour.

**Figure 7:**
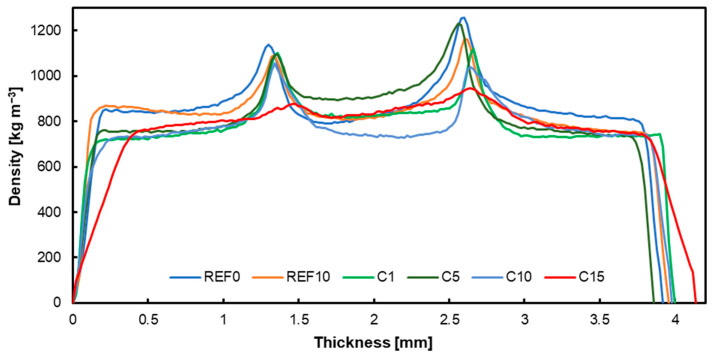
Density profile of tested panels with carbonized hazelnut shell flour.

The attached graphs below show the density profiles of the produced plywood, each with the respective fillers applied: native hazelnut shell flour (Figure 5), chemically modified hazelnut shell flour (Figure 6), carbonized hazelnut shell flour (Figure 7). Comparing the presented data, it is noticeable that particularly in the case of modified hazelnut shell dust, the joints with filler contents above 1% do not form characteristic peaks in the adhesive bond, which may also explain the unfavorable strength results. However, considering variants containing 1% alternative filler, it can be observed that the adhesive peaks are comparable to the reference values. According to the water absorption studies, activated carbon had the lowest absorbent properties. Consequently, more activated carbon resulted in greater plywood thickness with water absorbed by the wood. Generally, the more solid components there were, the greater the plywood thickness became [70]. The bond line is influenced by factors such as pH, buffer capacity, and the surface free energy of the perpendicular layer material [71,72].

### 3.5. Formaldehyde Emission

**Figure 8:**
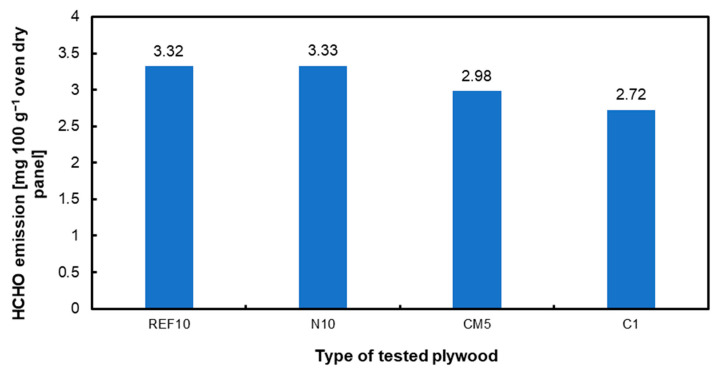
Formaldehyde emission of selected panels with various fillers of hazelnut shells.

The formaldehyde emissions for the different options are displayed in Figure 8. As the modification of hazelnut shell dust increases, its effectiveness in reducing formaldehyde emissions also improves. Additionally, the literature provides examples of other filler substitutes for plywood, such as chestnut bark meal and fir bark, which similarly contribute to lowering formaldehyde emissions in plywood production [73]. Beech bark has been utilized as a filler in urea–formaldehyde adhesives for plywood, reducing formaldehyde emissions by at least 46% [74]. The researchers demonstrated that bamboo-based activated carbon has a significantly higher formaldehyde removal rate than bamboo charcoal, highlighting the superior effectiveness of activated carbon in reducing formaldehyde emissions [75]. Furthermore, activated carbon has been demonstrated to reduce formaldehyde emissions effectively. Studies have shown that different forms of activated carbon, such as commercial activated carbon (CAC) and biomass-derived activated carbon, have good adsorption capability for formaldehyde [76,77,78].

## 4. Conclusions

This study confirmed the effective use of hazelnut shells as fillers in three-ply ply-wood production. The introduction of 10 pbw hazelnut shell flour significantly enhanced the modulus of rupture and modulus of elasticity, indicating the increased strength and flexibility of the material.

Chemically modified hazelnut shell flour showed optimal results at a 5 pbw concentration, demonstrating a beneficial impact on the mechanical properties of plywood. However, higher concentrations of chemically modified flour resulted in a deterioration of strength parameters. Carbonized flour derived from hazelnut shells, even at a 1 pbw concentration, significantly improved plywood’s bending strength. Carbon particles effectively reduced formaldehyde emissions and could improve water resistance, indicating better dimensional stability and a reduced environmental impact. The studies confirmed that excessive filler content negatively impacts the strength parameters of plywood. Optimizing the filler concentration is crucial for achieving the material’s best mechanical and operational properties.

The research indicates the practical feasibility of using hazelnut shell flour in plywood production instead of the currently commonly used cereal flour, which is a part of the food chain. This approach could revolutionize the wood materials industry by providing a greener and more efficient solution. Further research in that field can be focused both on the optimization of the use of tested fillers of various modifications as well as on the incorporation of complex research, including LCA, that should provide broader information about the potential application of modified or non-modified hazelnut shells flour.

The use of hazelnut shells as filler promotes sustainability and waste reduction. This solution supports a circular economy, reducing carbon emissions and encouraging the use of renewable resources.

The research demonstrates that hazelnut shells can be a valuable, eco-friendly additive in plywood production. Their use improves plywood’s mechanical and operational properties while reducing its environmental impact, supporting sustainability, and promoting the use of renewable resources.

## Figures and Tables

**Table 1:** Compositions of bonding mixtures and their curing time at 100 °C.

Variant Label	Filler Type	Filler Content	Curing Time [s]
		[pbw ^1^ per 100 pbw of Resin]	
REF0	-	0	88
REF10	Rye flour	10	86
N1	Native hazelnut shell flour	1	88
N5		5	88
N10		10	86
N15		15	84
CM1	Chemically modified hazelnut shell flour	1	87
CM5		5	85
CM10		10	84
CM15		15	82
C1	Carbonized hazelnut shell flour	1	86
C5		5	82
C10		10	80
C15		15	76

^1^ pbw—parts by weight.

**Table 2:** The statistical assessment results of mean values.

Test Type	Type of Tested Plywood													
	REF0	REF10	N1	N5	N10	N15	CM1	CM5	CM10	CM15	C1	C5	C10	C15
MOE	a ^1^	b	a	a	a, b	a	a	a	a	a	b	b	a, b	a
MOR	a	a, b	c	d	a	d	a	a	d	e	a, b	a	a	e
Shear strength	a	b	a	c	c	c	c	c	c	a, c	b	b, c	c	a, c

^1^ a–e homogenous groups.

## Data Availability

The data presented in this study are available in the open-access repository: https://doi.org/10.18150/WGJKJA (created and accessed on 27 July 2024).
